# Needle-injectable microcomposite cryogel scaffolds with antimicrobial properties

**DOI:** 10.1038/s41598-020-75196-1

**Published:** 2020-10-27

**Authors:** Kasturi Joshi Navare, Thibault Colombani, Mahboobeh Rezaeeyazdi, Nicole Bassous, Devyesh Rana, Thomas Webster, Adnan Memic, Sidi A. Bencherif

**Affiliations:** 1 Department of Chemical Engineering, Northeastern University, Boston, MA 02115 USA; 2 Wenzhou Institute for Biomaterials and Engineering, Wenzhou, 325001 China; 3 Center of Nanotechnology, King Abdulaziz University, Jeddah, 21589 Saudi Arabia; 4 Department of Bioengineering, Northeastern University, Boston, MA 02115 USA; 5 Sorbonne University, UTC CNRS UMR 7338, Biomechanics and Bioengineering (BMBI), University of Technology of Compiègne, 60203 Compiègne, France; 6 Harvard John A. Paulson School of Engineering and Applied Sciences, Harvard University, Cambridge, MA 02138 USA

**Keywords:** Biotechnology, Microbiology, Medical research, Engineering, Materials science

## Abstract

Porous three-dimensional hydrogel scaffolds have an exquisite ability to promote tissue repair. However, because of their high water content and invasive nature during surgical implantation, hydrogels are at an increased risk of bacterial infection. Recently, we have developed elastic biomimetic cryogels, an advanced type of polymeric hydrogel, that are syringe-deliverable through hypodermic needles. These needle-injectable cryogels have unique properties, including large and interconnected pores, mechanical robustness, and shape-memory. Like hydrogels, cryogels are also susceptible to colonization by microbial pathogens. To that end, our minimally invasive cryogels have been engineered to address this challenge. Specifically, we hybridized the cryogels with calcium peroxide microparticles to controllably produce bactericidal hydrogen peroxide. Our novel microcomposite cryogels exhibit antimicrobial properties and inhibit antibiotic-resistant bacteria (MRSA and *Pseudomonas aeruginosa*), the most common cause of biomaterial implant failure in modern medicine. Moreover, the cryogels showed negligible cytotoxicity toward murine fibroblasts and prevented activation of primary bone marrow-derived dendritic cells ex vivo. Finally, in vivo data suggested tissue integration, biodegradation, and minimal host inflammatory responses when the antimicrobial cryogels, even when purposely contaminated with bacteria, were subcutaneously injected in mice. Collectively, these needle-injectable microcomposite cryogels show great promise for biomedical applications, especially in tissue engineering and regenerative medicine.

## Introduction

The goals of tissue engineering are centered on the repair or replacement of diseased or damaged tissue by using a combination of scaffolds, cells, and signaling (i.e., biochemical and biophysical) cues^1–5^. Three-dimensional (3D) scaffolds are designed to provide a structural support and physical environment for cell attachment and subsequent tissue development^6–9^. Various biomaterials have been utilized to fabricate bioactive scaffolds with tunable physicochemical properties (e.g., rigidity, elasticity, and biodegradation) while exhibiting remarkable biocompability^10–13^. For instance, hydrogels, cross-linked networks made from synthetic and/or naturally occurring hydrophilic polymers, absorb and hold large amounts of water while retaining their 3D integrity and mimicking the native cellular microenvironment^14–26^. To match the mechanical properties of human tissues, from soft to more rigid, several strategies have been investigated to tune gel stiffness^10,27,28^. Amongst the methods available are (*i*) adjusting the cross-linking density (e.g., cross-linking agents, polymer concentration), (*ii*) implementing a double network hydrogel system (e.g., interpenetrating polymer network), and (*iii*) hybridizing hydrogels with composite materials (e.g., carbon nanotubes, graphene oxide)^10,29–31^.

Recently, injectable hydrogels, such as shear-thinning or in situ gelling hydrogels, have been developped to prevent trauma and other complications associated with the invasive nature of surgical implantations^28,32,33^. Yet, these non-invasive hydrogels face a number of challenges, including the delivery of large preformed hydrogels and the control over their microstructural features (i.e., pore size) to facilitate cellular infiltration and molecular diffusion of proteins, oxygen, and nutrients/waste products^28,33–36^. To address these challenges, cryogels, a type of macroporous hydrogels with unique properties, have been fabricated via cryopolymerization at subzero temperatures. These gels have large macroscale interconnected pores and improved mechanical properties compared to their mesoporous hydrogel counterparts^37–43^. These architectural features can allow easier nutrient inflow and waste removal, both of which are important in tissue engineering applications^42,44^. Furthermore, the highly dense polymer walls and large pores allow cryogels to undergo reversible compression (i.e., up to 90% of the initial volume)^37,42,44,45^. These cryogels have shape-memory properties and can undergo injection through hypodermic needles without any structural damage, allowing their minimally invasive delivery into the body^17,33,46,47^. A variety of biopolymers have been used to fabricate biomimetic cryogels for tissue engineering applications^48–53^.

Over the past years, biomaterials made from extracellular matrix (ECM) components have been used to better recapitulate native tissue microenvironments^54^. One component of the ECM and an important constituent of synovial fluid is hyaluronic acid (HA), a linear polysaccharide made from unbranched recurring components of N-acetylglucosamine and glucuronic acid. Furthermore, HA has been shown to play an important role in several cellular processes, such as wound healing and angiogenesis^30,55^. Biomaterial scaffolds made from HA precursors have been used to engineer various tissues^56,57^. HA-based biomaterials are generally nonimmunogenic, promote long-term water retention, and exhibit good flexibility^56,58^. However, the growing incidence of antibiotic-resistant infections remains a major challenge when designing implantable biomaterials^37,59,60^.

The U.S. Centers for Disease Control and Prevention (CDC) reported that over 100,000 biomaterial and surgical site infections occurred in 2011 alone^61^. Common biomedical implant-related pathogens include *Staphylococcus aureus* (*S. aureus*), *Escherichia coli* (*E. coli*), and *Pseudomonas aeruginosa* (*P. aeruginosa*). Complications from implant-related infections can impede tissue regeneration, delay wound healing^59^, and induce the development of biofilms that can require > 1000 times higher doses of antibiotics for effective treatments^16,62–64^. To address this challenge, diverse strategies have been deployed to prevent microbial contamination of biomaterials. Some of the strategies have relied on developing antibiotic-containing surface coatings or incorporating antimicrobial peptides within biomaterial constructs^46,62,64–69^. However, a number of limitations of these approaches still remain. For example, the use of antiadhesive coatings can hinder cell attachment and lead to poor tissue integration. Similarly, the overuse of antimicrobial peptides poses the risk of increased bacterial resistance^47,66,67,70^. Zhao et al. developed antimicrobial cryogels fabricated from chitosan^70^. However, scaffolds made with polycations such as chitosan are known to be proinflammatory with slow in vivo degradation^71,72^. Adverse host immune reactions of biomaterials can impair healing and result in implant isolation (e.g., fibrotic encapsulation) and ultimately rejection^73^. To address this shortcoming, researchers have turned to the incorporation of nanomaterials such as metal and metal oxide nanoparticles^74,75^ to improve the antibacterial activity of cryogels. However, the incorporation of metallic nanoparticles has also been associated with decreased mammalian cell proliferation and viability^76,77^. For instance, Chaturvedi et al. incorporated antimicrobial zinc oxide (ZnO) nanoparticles into polyvinyl alcohol cryogels and studied their biocompatibility^78^. They reported that at high concentration, ZnO nanoparticles can induce hemolysis, a condition leading to the breakdown of red blood cells. Similarly, Zou et al. incorporated silver (Ag) nanoparticles into chitosan-poly(ethylene glycol) cryogels^69^. Even though their composite cryogels exhibited improved antibacterial activity, the effect of Ag nanoparticles on mammalian cells was not studied.

One recent alternative strategy is the development of nanocomposite biomaterials that have intrinsic antimicrobial properties. Recently, Zhao et al. developed nanocomposite cryogels that had antibacterial properties and could be used for non-compressible hemorrhage and wound healing^70^. Specifically, they fabricated a group of carbon nanotube-reinforced chitosan-based cryogels that were syringe injectable and conductive. However, their platform was not able to be delivered using minimally invasive methods (i.e., needle-injectable) and was built using polycations that are generally reported to be proinflammatory in vivo^71^. Furthermore, quaternized chitosan has limited in vivo degradation. Another example was reported by Wang et al. 2010, who developed antimicrobial calcium peroxide (CP)-incorporated polycaprolactone nanofibers. These CP particles can release oxygen and other oxygen species to support tissue engineering and wound healing platforms^63,79,80^. Specifically, CP dissociates to form calcium hydroxide and hydrogen peroxide (Eq. 1), which can further decompose and lead to the release of hydroxyl ions (Eq. 2), elevation of pH, and free radical-mediated antifungal^81^, antiviral^82^, and antibacterial effects^83–85^.

1 $${\text{CaO}}_{2} + 2{\text{H}}_{2} {\text{O}} \to {\text{Ca}}\left( {{\text{OH}}} \right)_{2} + {\text{H}}_{2} {\text{O}}_{2}$$

2 $${\text{Ca}}\left( {{\text{OH}}} \right)_{2} \to {\text{Ca}} \downarrow +\:2{\text{OH}}^{ - } \;{\text{and}}\;{\text{H}}_{2} {\text{O}}_{2} \to {\text{OH}}^{ - } +\:{\text{OH}} \bullet$$

**Figure 1:**
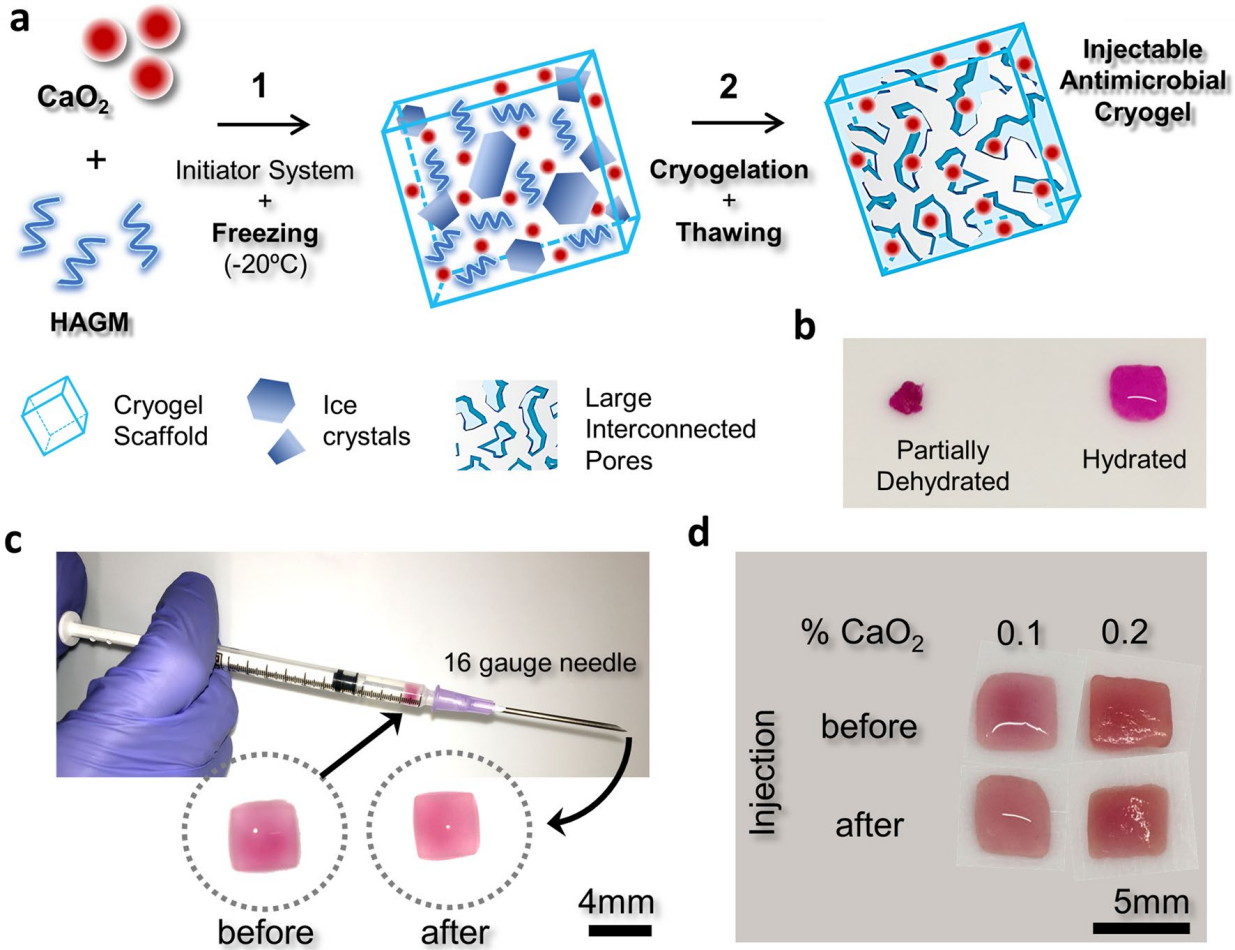
Engineering antimicrobial and injectable microcomposite cryogels. (**a**) Overview of the fabrication process of antimicrobial CP-containing injectable cryogels: (*1*) cryogels were fabricated using 4% HAGM with different amounts of CP (0–0.2% CaO_2_); an initiator system (APS/TEMED) is added to an aqueous HAGM solution prior to cryopolymerization at − 20 °C. (*2*) Cryotreatment involves phase separation with ice crystal formation, cross-linking and gelation. Thawing of ice crystals (porogens) results in an interconnected macroporous cryogel network. (**b**) Cryogel partially dehydrated over Kimwipe regains its original shape and size after hydration. HAGM cryogels were stained with rhodamine for visualization. (**c**) Following injection through a 16G hypodermic needle, cryogels regain their original shape and dimensions. (**d**) Cryogels retain their encapsulated CP after needle injection as indicated by the Alizarin Red S staining (n = 5).

In this study, we fabricated antimicrobial cryogels using methacrylated hyaluronic acid (HAGM) hybridized with CP microparticles at different concentrations (Fig. 1). These microcomposite cryogels exhibited a highly interconnected and dense polymer network that can collapse and be injected through a 16G needle without any mechanical damage (Supplementary Movies 1 and 2). We investigated changes in the physical properties of these cryogels as a result of CP incorporation. Specifically, we determined the effects of CP inclusion on cryogel pore size distribution, interconnectivity, swelling, and mechanical properties. We monitored the release of hydrogen peroxide from CP present within the cryogel scaffolds and how the concentration of CP within the cryogels affected their antibacterial activity. We assessed the antibacterial activity using some of the most virulent bacterial pathogens (i.e., superbugs), including methicillin-resistant *Staphylococcus aureus* (MRSA) and *P. aeruginosa*. Next, we assessed the cytocompatibility of cryogels using NIH/3T3 mouse fibroblasts (i.e., cell viability) and the in vitro immunogenic response (i.e., dendritic cell activation) using both CP-free and CP-containing cryogels. Finally, the in vivo biodegradation and immunogenic response across the cryogels were evaluated using C57BL/6 mice.

## Results

### Characterization of cryogels

**Figure 2:**
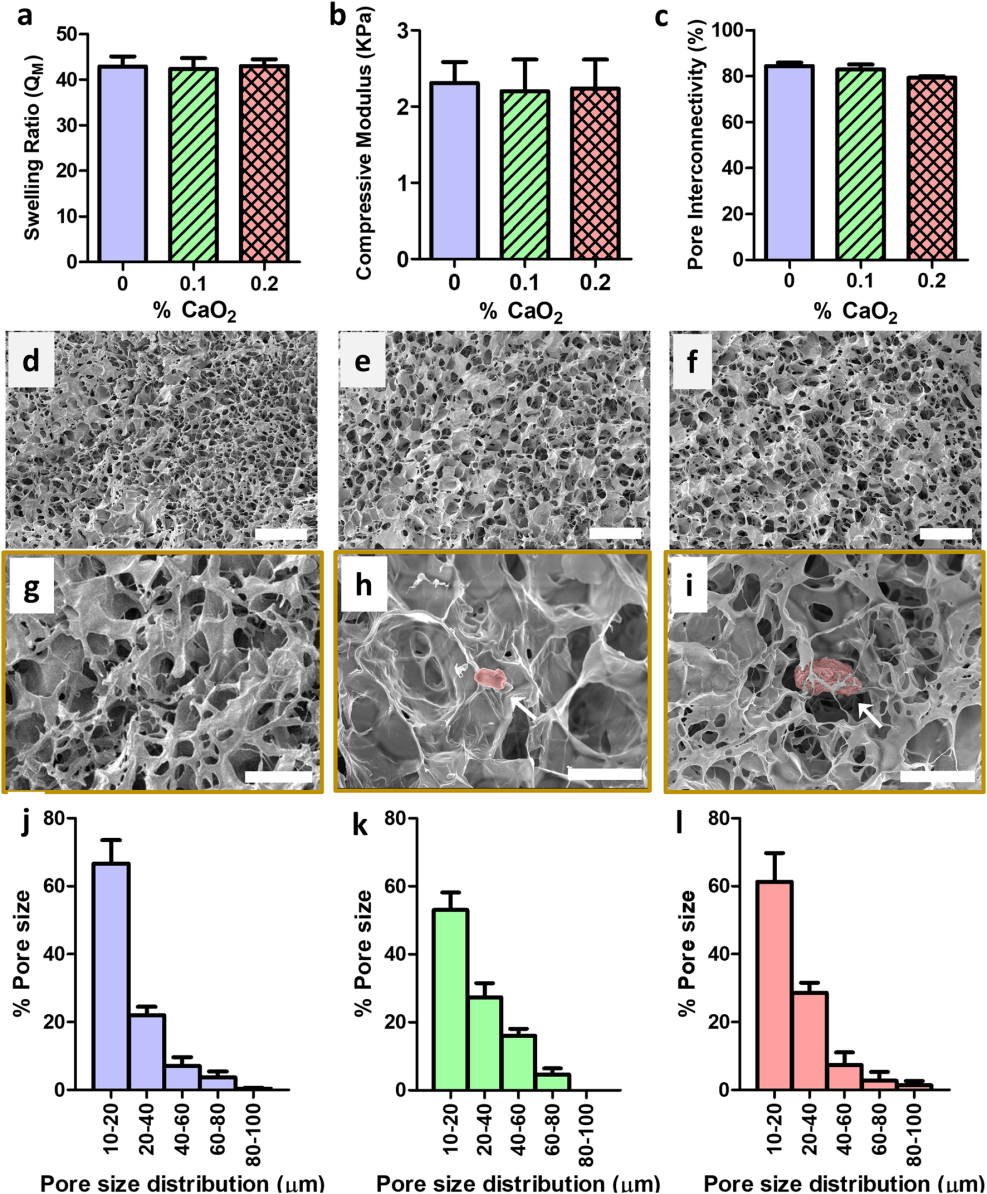
Antimicrobial cryogels have advantageous mechanical properties and microarchitectural features. A total of three types of cryogels were fabricated using 4% HAGM and different amounts of CP (0–0.2% CaO_2_). (**a**) swelling ratio, (**b**) Young’s moduli, and (**c**) evaluation of pore connectivity. Cross-sectional SEM images showing interconnected macroporous network of (**d**) CP-free cryogels (0% CaO_2_), (**e**) CP-containing (0.1% CaO_2_) cryogels, and (**f**) CP-containing (0.2% CaO_2_) cryogels. SEM samples (n = 3) were scanned and representative images have been presented. Cross-sectional SEM images for visualization of CP (pseudocolored in pink): (**g**) CP-free cryogel (0% CaO_2_), (**h**) CP-containing (0.1% CaO_2_) cryogel, and (**i**) CP-containing (0.2% CaO_2_) cryogel. Pore size distribution histograms of (**j**) CP-free cryogels (0% CaO_2_), (**k**) CP-containing (0.1% CaO_2_) cryogels, and (**l**) CP-containing (0.2% CaO_2_) cryogels. Scale bars = 100 µm (d, e, and f) and 50 µm (g, h, and i).

We first characterized the mechanical and structural properties of the fabricated cryogels. Specifically, we examined three cryogel formulations, the first without any CP (i.e., 0% CaO_2_); the second with 0.1% (w/v) CP (i.e., 0.1% CaO_2_); and the third prepared with a 0.2% (w/v) CP (i.e., 0.2% CaO_2_). We first assessed how the cryogel swelling ratio varied as a function of CP concentration. We observed that all cryogel swelling ratios ranged from Q_M_ = 41 to 45, remaining relatively unchanged regardless of the amount of CP incorporated (Fig. 2a). Next, we measured the compressive moduli for each cryogel formulation. The comparison of the compressive moduli revealed that all cryogels exhibited low compressive moduli of approximately 2.2 kPa (Fig. 2b). Similarly, the pore interconnectivity of the cryogels was high (i.e., ~ 80%) and remained similar across the investigated CP concentrations (Fig. 2c).

We next characterized the morphological features of the cryogels. Scanning electron microscopy (SEM) imaging show that after cryogelation, a network of large interconnected pores remains (Fig. 2d–f). Overall, the cryogels displayed a continuous, uniform network of interconnected macropores, with the presence of solid CP particles (0.1–0.2% CaO_2_) embedded within the polymer network (Fig. 2 h,i). Finally, the average pore sizes ranged from 25 to 30 µm (Fig. 2 g–i) with a similar pore size distribution patterns (Fig. 2j–l) among all three cryogel formulations. Data are presented as the mean ± standard error of the mean (n = 4).

### Kinetics of hydrogen peroxide release from cryogels

**Figure 3:**
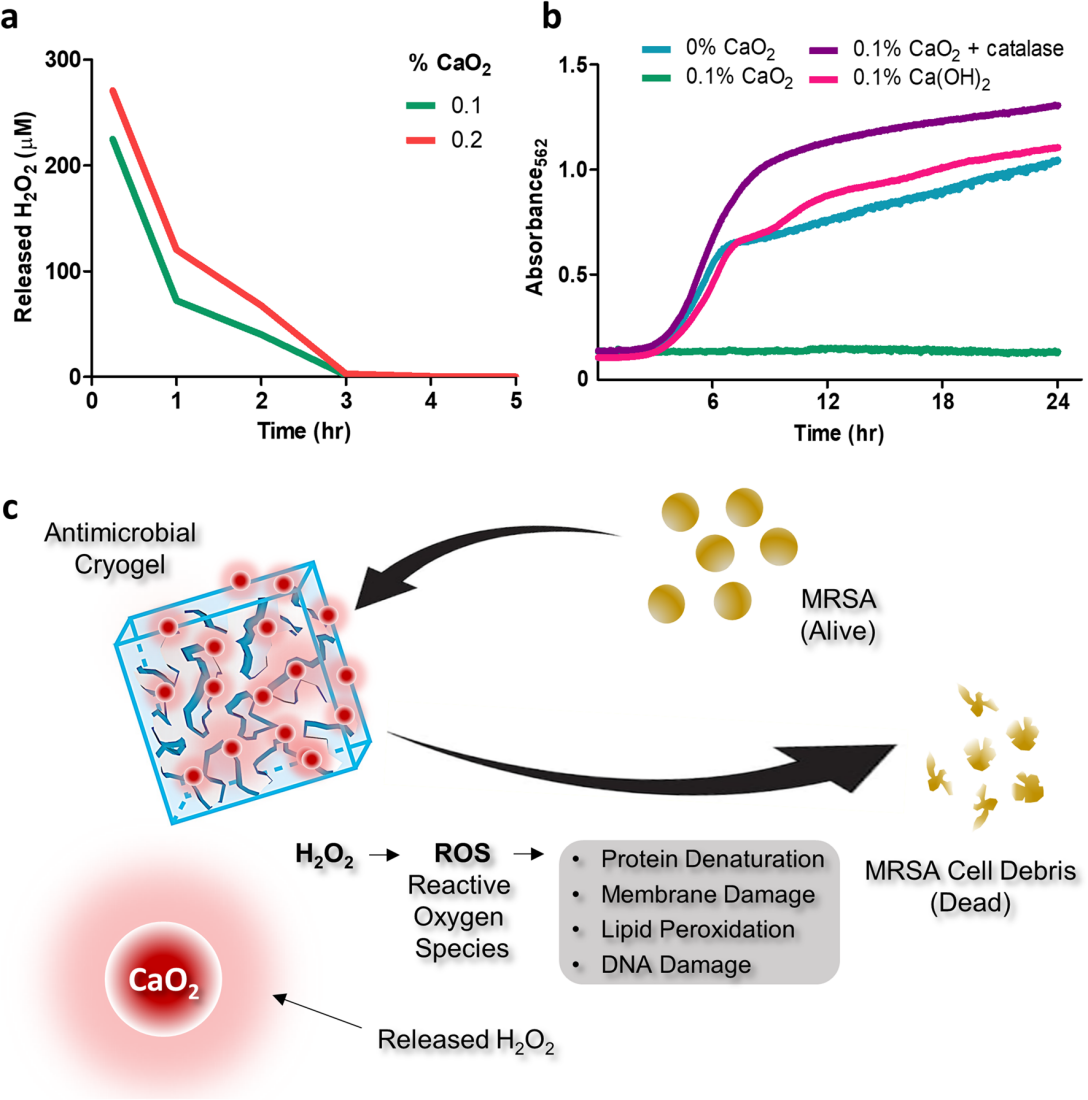
CP-containing cryogels generate antimicrobial hydrogen peroxide (H_2_O_2_). (**a**) Release of H_2_O_2_ from the cryogels following injection through a 16G needle, n = 3. (**b**) Growth monitoring absorbance values of MRSA after 24 h of incubation with CP alone, CP with catalase and Ca(OH)_2_ along with a control (CP-free medium), average values of three experiments have been plotted. (**c**) Schematic representing the biocidal action of CP-containing cryogels against bacteria.

Hydrolysis of the CP particles entrapped within the cryogels leads to the formation of hydrogen peroxide. The release profile of hydrogen peroxide within the first 5 h was compared between the two (i.e., 0.1% vs 0.2% CaO_2_) square-shaped cryogels (dimensions: 4 mm × 4 mm × 1 mm). Both cryogel variants showed similar release patterns with over 60% release within the first 15 min followed by a progressively decreasing release pattern within the next 3 h. The 0.1% CaO_2_ cryogels had a cumulative hydrogen peroxide release of 331 µmol, while for the 0.2% CaO_2_ cryogels, the cumulative amount of hydrogen peroxide released reached 468 µmol (Fig. 3a).

Another byproduct of CP hydrolysis is calcium hydroxide (i.e., Ca(OH)_2_) precipitate which has been reported to have limited antibacterial properties. We next compared the effect of hydrogen peroxide and calcium hydroxide on MRSA growth. The bacterial growth was monitored for 24 h using culture broth containing CP, CP supplemented with bovine catalase, and calcium hydroxide. The role of catalase was to break down hydrogen peroxide into water and oxygen, and as a result, neutralizing its antibacterial effect^86^. We observed bacterial growth inhibition only in the presence of CP, while MRSA grew unhindered under the other conditions. This result suggests that the antibacterial activity of CP is predominantly based on hydrogen peroxide activity and that calcium hydroxide had minimal or no effect on bacteria (Fig. 3b,c).

### Antibacterial activity of microcomposite CP-containing cryogels

**Figure 4:**
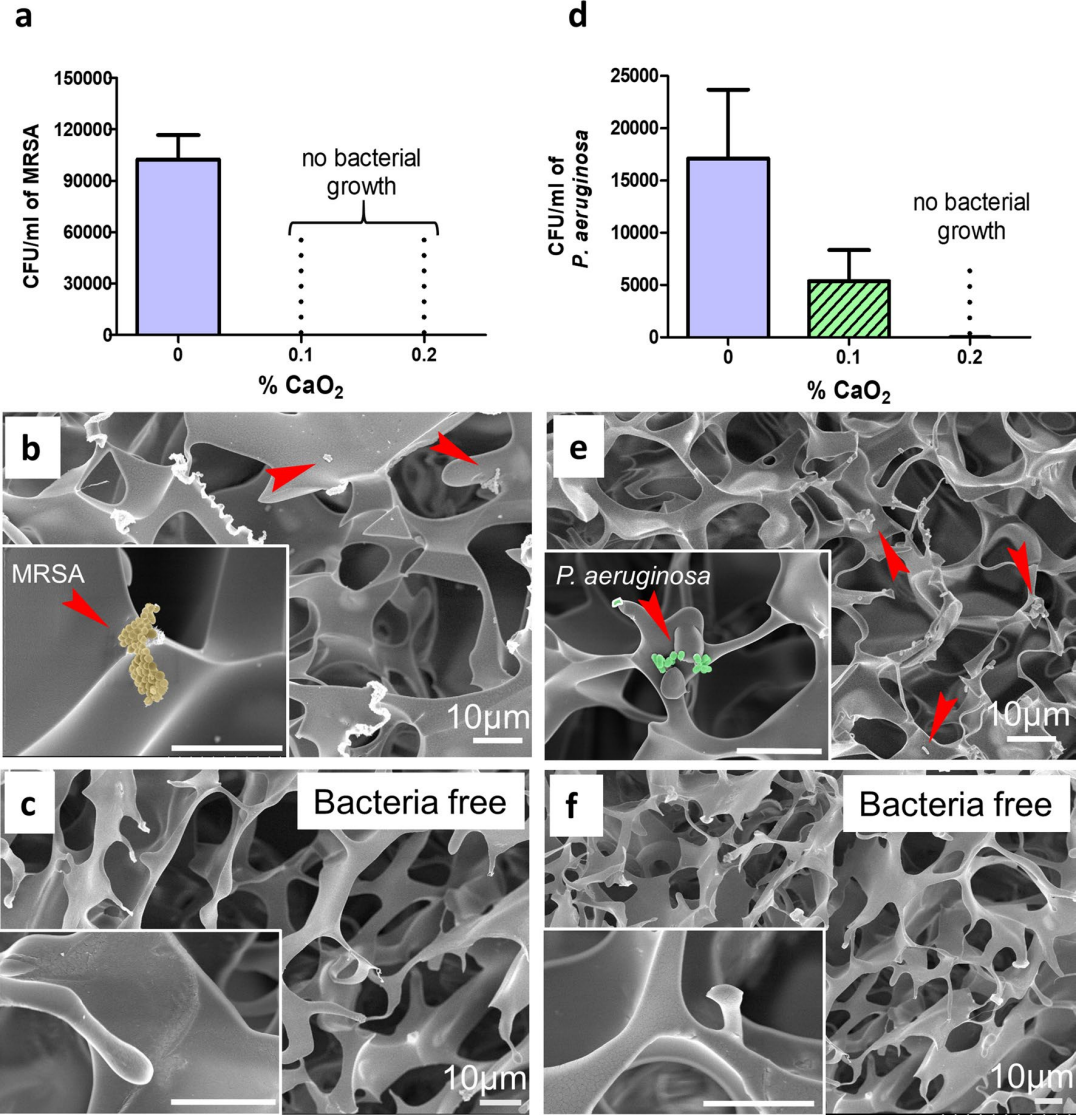
Antibacterial activity of CP-containing microcomposite cryogels. (**a**) Residual CFU i.e., colony forming units per ml of MRSA after 6 h of contact period within different cryogels; namely CP-free cryogels (0% CaO_2_), CP-containing (0.1% CaO_2_) cryogels, and CP-containing (0.2% CaO_2_) cryogels. (**b**) Cross-sectional SEM of CP-free cryogels (0% CaO_2_), and (**c**) CP-containing (0.1% CaO_2_) cryogels. (**d**) Residual CFU/ml *P. aeruginosa* after 6 h of contact period across different cryogels. (**e**) cross-sectional SEM of CP-free cryogel (0% CaO_2_), and (**f**) CP-containing (0.2% CaO_2_) cryogels. Data are representative of three independent experiments and are presented as the mean ± standard error of the mean (n = 4). For visualization, MRSA and *P. aeruginosa* were pseudocolored in yellow and green, respectively. Scale bars = 10 µm.

Two pathogens (i.e., MRSA and *P. aeruginosa*) were used to study the antibacterial properties of cryogels hybridized with CP. First, partially dehydrated cryogels were contaminated with a known density of bacteria [i.e., colony forming units (CFUs)]. After 6 h of bacterial incubation within the cryogels, the number of CFUs was quantified. Figure 4a–c shows the trend observed for MRSA incubated with CP-free and CP-containing cryogels. In the absence of CP particles, bacteria within the cryogels not only remained viable but also underwent growth and expansion. On the other hand, cryogels containing 0.1–0.2% CP exhibited total bacterial growth inhibition. Similar findings were observed in the case of *P. aeruginosa* (Fig. 4d–f). For example, in the case of CP-free (0% CaO_2_) cryogels, the number of CFUs significantly increased, whereas cryogels containing 0.2% CP displayed total bacterial inhibition. However, cryogels containing 0.1% CP were not as effective against *P. aeruginosa* as they were with MRSA, showing that approximately half of the *P. aeruginosa* remained viable after 6 h (Supplementary Figs. 1 and 2). The antibacterial activity of CP-containing cryogels was also further tested against other multi-drug resistant bacteria, namely *E. coli* and *Streptococcus pyogenes* (Supplementary Figs. 3 and 4).

To visually check for the presence of bacteria, cryogels were fixed and imaged by SEM. Figure 4b shows the presence of MRSA within CaO_2_-free cryogels. Conversely, Fig. 4c depicts the absence of bacteria within cryogels containing 0.1% CP. Similarly, the presence of *P. aeruginosa* can be seen in CaO_2_-free cryogels (Fig. 4e) but was absent for cryogels containing 0.2% CP (Fig. 4f), clearly demonstrating the antibacterial potential of microcomposite CP-containing cryogels.

### In vitro cytocompatibility studies of antimicrobial cryogels

**Figure 5:**
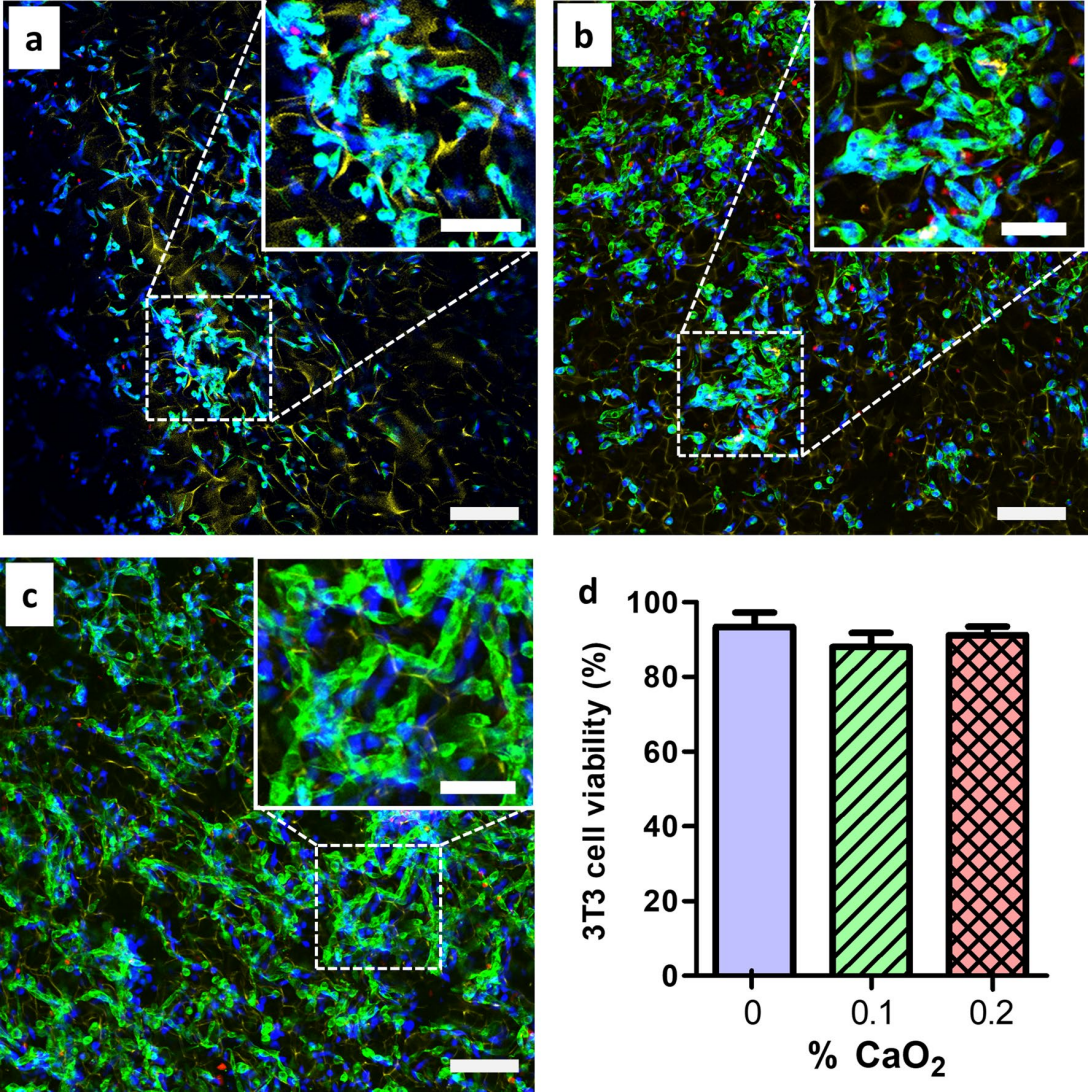
Antimicrobial CP-containing cryogels are cytocompatible towards murine fibroblasts*.* Confocal images showing mouse embryonic fibroblast NIH/3T3 cells cultured for 24 h in three CP-containing HAGM cryogels: (**a**) 0%, (**b**) 0.1%, and (**c**) 0.2% CaO_2_. Red color depicts dead cells, blue color represents cell nuclei, green color highlights cell cytoskeleton and yellow color shows the polymer network of cryogels. (**d**) Evaluation of NIH/3T3 cell viability (%). Scale bars = 200 µm (large confocal images) and 100 µm (inset confocal images). Data are presented as the mean ± standard error of the mean (n = 4).

We next evaluated the cytotoxicity of the antibacterial cryogels using mouse fibroblasts NIH/3T3 cells. Specifically, after 24 h of incubation, cell attachment to the cryogels was observed by confocal microscopy. All CP-free and CP-containing cryogels (0.1–0.2% CaO_2_) supported the proliferation and attachment of cells along the polymer walls (Fig. 5a–c). We observed high cell viability (i.e., ~ 90%) for all cryogels (Fig. 5d). These findings indicate that antibacterial cryogels release enough hydrogen peroxide to kill bacterial pathogens but that the level released is below the toxicity threshold of a commonly used mammalian cell line.

### In vitro immunogenic response of antimicrobial cryogels

**Figure 6:**
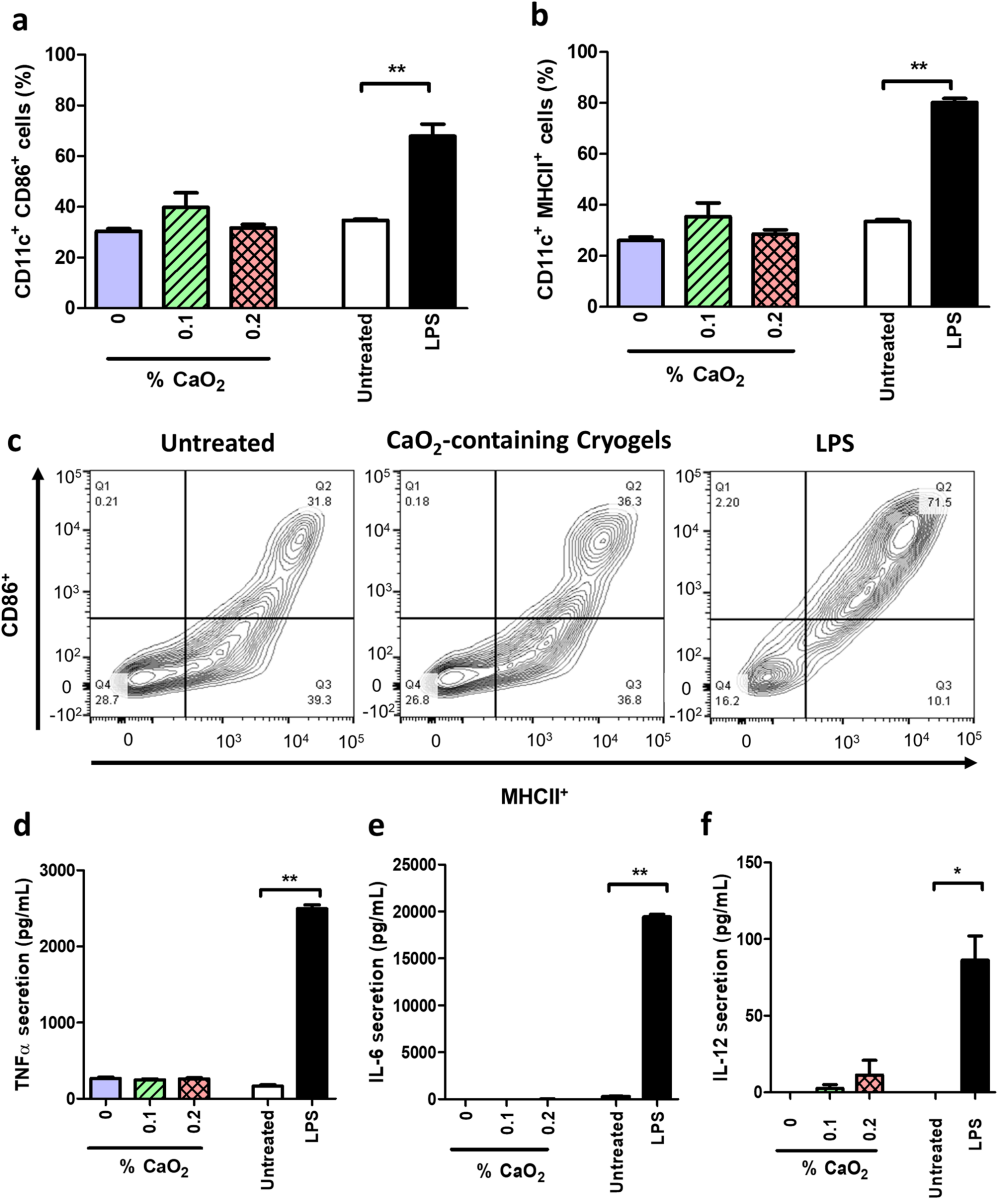
Antimicrobial CP-containing cryogels do not activate BMDCs. BMDCs were cultured for 24 h in the following conditions: in the presence of CP-free cryogels (0% CaO_2_), CP-containing (0.1% CaO_2_) cryogels, CP-containing (0.2% CaO_2_) cryogels, cryogel-free medium (untreated, negative control) and LPS-containing medium (100 ng/mL, positive control). Fractions of activated (**a**) CD11c^+^ CD86^+^ BMDCs and (**b**) CD11c^+^ MHCII^+^ BMDCs after 24 h of incubation in the different conditions. (**c**) Expression profile of CD86 and MHCII in CD11c^+^ DCs. The results shown are representative of 4 independent experiments. Concentrations of DC-secreted (**d**) TNF-α, (**e**) IL-6, and (**f**) IL-12 cytokines from cell culture supernatants after 24 h of incubation in the different conditions. Data are presented as the mean ± standard error of the mean (n = 5) and were analyzed using one-way analysis of variance (ANOVA) and the Dunnett′s post-hoc test (*p < 0.05, **p < 0.01, compared to the untreated condition).

Dendritic cells (DCs) are antigen-presenting cells that can play a crucial role in mounting an effective immune response. They secrete an array of proinflammatory cytokines, some of which are responsible for T cell differentiation^76,77^. Immunogenicity with bone marrow-derived DCs (BMDCs) was assessed using all three cryogel formulations. We further compared BMDC stimulation with cryogel-free (negative control) and lipopolysaccharide (LPS)-containing (positive control) media. Fractions of activated CD11c^+^CD86^+^ and CD11c^+^MHCII^+^ BMDCs were measured by immunostaining in conjunction with fluorescence-activated cell sorting (FACS) analysis. CP-containing cryogels induced basal expression levels of CD86 and MHCII receptors nearly equivalent to those induced by the negative control (Fig. 6a–c). Finally, we characterized cell culture supernatants by ELISA for the DC-mediated secretion of several proinflammatory cytokines. In good agreement with the minimally activated DCs, the depicted low concentrations of IL-6, IL-12 and TNF-α (Fig. 6d–f) further demonstrate that all cryogels induced minimal immunogenicity.

### In vivo immunogenic response and biodegradation of antimicrobial cryogels

**Figure 7:**
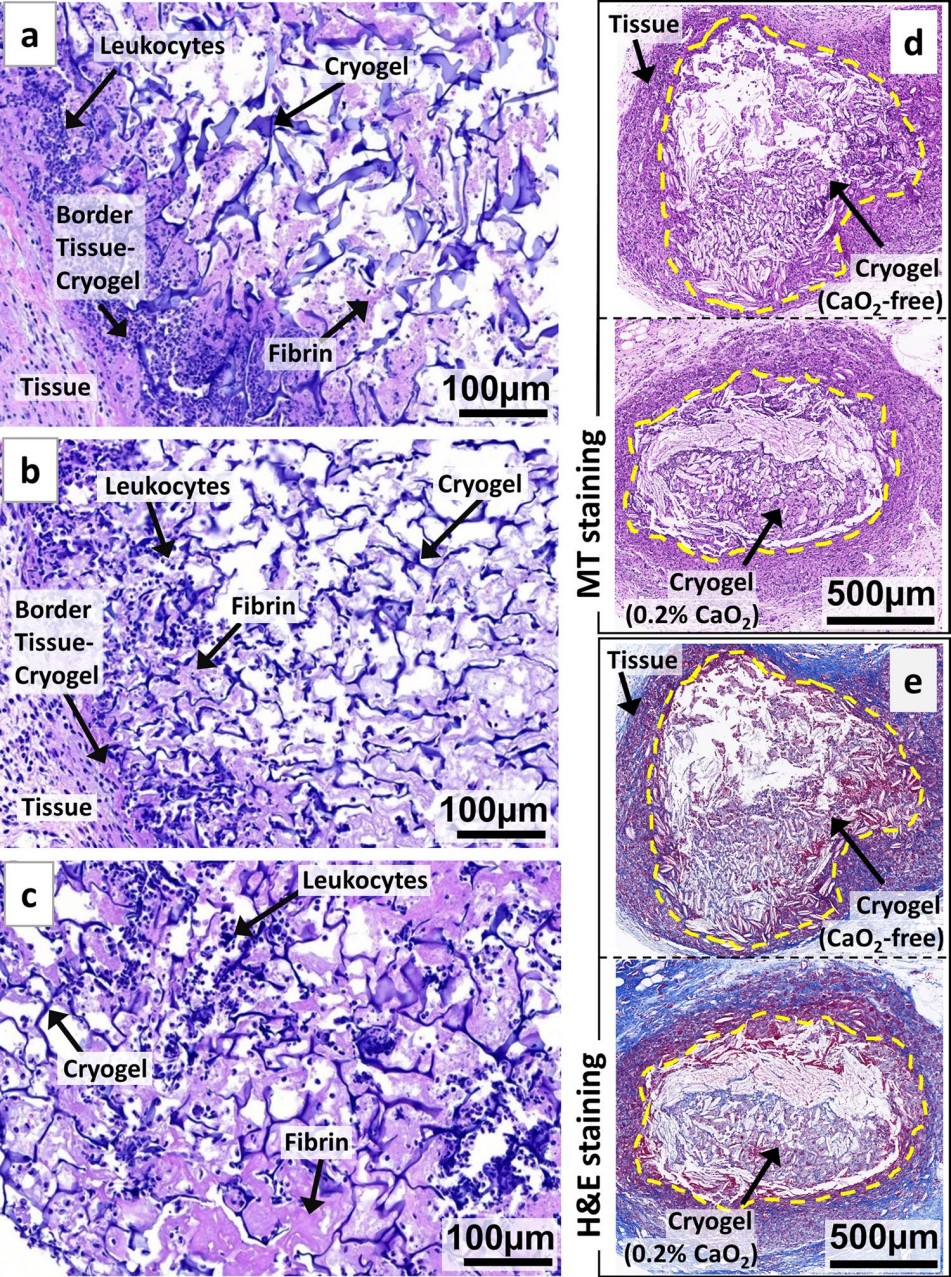
Antimicrobial microcomposite cryogels are biodegradable and elicit minimal host inflammatory responses. H&E staining of HAGM cryogel scaffold sections explanted 4 days following subcutaneous injections in the dorsal flanks of C57BL/6 mice: (**a**) CP-free cryogel (0% CaO_2_), (**b**) CP-containing (0.1% CaO_2_) cryogel, and (**c**) CP-containing (0.1% CaO_2_) cryogel contaminated with *P. aeruginosa*. H&E staining highlights the macroporous polymeric network of cryogels (interconnected dark blue fibers), infiltrated leukocytes (dark blue dots), fibrin formation (purple), and surrounding tissues (cryogel-free). Masson's trichrome (MT) (**d**) and H&E (**e**) staining of square-shaped implants explanted 2 months following subcutaneous injections in the dorsal flanks of C57BL/6 mice: CP-free (0% CaO_2_) and CP-containing (0.2% CaO_2_) cryogels (dimensions: 4 mm × 4 mm × 1 mm). The yellow doted lines indicate the boundary between the cryogel and the host tissue. Images are representative of n = 5 samples per condition.

Finally, we examined the immunological response of the antimicrobial cryogels in a mouse model. CP-free cryogels, CP-containing (0.1% CaO_2_) cryogels, and CP-containing (0.1% CaO_2_) cryogels contaminated with *P. aeruginosa,* were subcutaneously injected into the backs of C57BL/6 mice. Next, the cryogels were explanted on day 4 and subsequently stained with hematoxylin and eosin (H&E) for histological analysis (Fig. 7a–c). We assessed the cellular infiltration into cryogels as well as their integration within the surrounding tissues. Overall, across the three groups tested, cryogels were surrounded by a thin capsule of fibrin and induced a minimal infiltration of leukocytes (e.g., neutrophils and macrophages). It is worth noting that fibrin production and leukocyte infiltration were slightly more observable for the bacteria-contaminated antimicrobial cryogels. Consistent with their high degree of cytocompatibility and minimal in vitro DC activation, these microcomposite antimicrobial cryogels indicated minimal host inflammatory responses in mice.

The biodegradation of biomaterials is an essential criterion when designing implantable medical devices. Therefore, we have assessed the extent of cryogel degradation upon subcutaneous injection in mice. CP-free (0% CaO_2_) and CP-containing (0.2% CaO_2_) cryogels were tested and after a 2-month implantation, the explanted cryogels displayed signs of degradation (Fig. 7). Compared to their initial dimensions (4 mm × 4 mm × 1 mm), both types of cryogels were clearly smaller, especially for CP-containing cryogels (Fig. 7d–e). This set of data suggests that the incorporation of CP into cryogels may promote biodegradation.

## Discussion

Three-dimensional scaffolds that exhibit a highly porous architecture could be very useful for several biomedical applications especially in tissue engineering^1,75^. Macroporous cryogels have been made from a variety of natural polymers meant to recapitulate the composition and structural properties of the ECM^1,37,44^. For example, by varying the polymerization temperature and cooling rate^87^, the mechanical properties of cryogels could be tuned to match those of the native tissues. Similarly, injectable cryogels with improved mechanical properties compared to their hydrogel counterparts can be fabricated^87,88^. However, microbial infections remain a major challenge associated with scaffolds and biomedical implants. Specifically, in clinical orthopedics, complications related to pathogenic bacterial colonization represent a major barrier to tissue repair and healing^89^. Although various approaches have been explored to confer hydrogels with antimicrobial properties, they have been associated with a number of limitations, including cytotoxicity and poor tissue integration^90–92^. Here, we report the fabrication of multifunctional cryogels (i.e., needle-injectable, biodegradable, and with antimicrobial activity) that could improve current strategies for developing scaffolds for tissue engineering.

In our study, we incorporated CP that imparts antibacterial activity to the cryogel scaffolds. The hydrolysis of CP leads to the formation of calcium hydroxide and hydrogen peroxide. Although calcium hydroxide is used as an antimicrobial medication in dentistry^83^, it did not appear to drive the antibacterial activity of CP-containing cryogels at the concentration reported in our study (Fig. 3b). The presence of catalase (i.e., an enzyme that degrades hydrogen peroxide) resulted in the total loss of the antibacterial activity of CP, pointing to the production of hydrogen peroxide as the primary driver of the observed antibacterial effects. Conversely, in medical settings, hydrogen peroxide is used as an antiseptic solution for wound disinfection and irrigation at concentrations as high as 30%. Lower concentrations of 3–6% also have bactericidal action^93^. Unlike cryogels that are loaded with antibiotics^94,95^, our cryogels based on hydrogen peroxide-mediated bacterial inhibition have a minimal risk of eliciting resistance^96^. Although bacteria possess multiple antioxidant defenses to fight reactive oxygen species^86^ our CP-containing cryogels can release > 450 µmol of hydrogen peroxide producing hydroxyl radicals that cannot be detoxified by bacterial enzymes^86,96,97^.

Previously, scaffolds loaded with CP have been used for oxygen release^98^. Converting hydrogen peroxide from CP to water and oxygen can be useful for tissue engineering applications^99,100^. However, the concentration of CP has to be finely tuned to prevent cytotoxic side effects. In our study, CP-containing cryogels producing hydrogen peroxide were sufficient to inhibit bacterial growth, while there was minimal to no observable toxicity to mammalian cells. We showed that NIH/3T3 cells cultured with CP-containing cryogels exhibited high cell viability (Fig. 5). Similarly, these cryogels seemed unlikely to induce an inflammatory response^101,102^. We observed that these cryogels do not activate or trigger the secretion of proinflammatory cytokines when cultured with primary DCs (i.e., BMDCs). Similarly, in the in vivo setting, the cryogels induced minimal inflammatory reactions even when purposely contaminated with bacteria prior to implantation. This result reaffirms our hypothesis that CP-containing cryogels can provide strong antimicrobial activity and prevent bacterial colonization. In addition to its antimicrobial activity, hydrogen peroxide has been reported to have antiviral and antifungal properties^81,82^. We hypothesize that CP-containing cryogels could potentially have some biocidal actions against other microorganisms (e.g., virus, yeasts, molds) and further studies are needed. Another advantage of this platform is that the injectable scaffolds are preformed, enabling them to recover their shape at the site of injection and thus eliminating the need for in situ gelation. Furthermore, CP-containing cryogels exhibited signs of biodegradation after 2 months when subcutaneously injected in mice. The degradation may be the result of enzymatic hydrolysis and oxidation^88,89^. These unique characteristics make these microcomposite cryogels very attractive for a wide range of biomedical applications, including designing scaffolds for tissue engineering and regenerative medicine.

Multiple CP-containing cryogels were formulated for this study and the resulting concentration of microparticles was relatively low. While for the purpose of our study (i.e., to prevent implant-associated infections), the transient antimicrobial activity of our cryogels is acceptable, a more prolonged biocidal action may be required for other applications such as wound healing. However, a finely tuned balance needs to be achieved between hydrogen peroxide generation and its possible cytotoxicity on mammalian cells. To that end, adjusting CP hydrolysis, and ultimately hydrogen peroxide production within its biocompatible range, is necessary. Using other metal peroxides such as magnesium peroxide could potentially provide a more sustained yet low level of hydrogen peroxide release. Although it may be not needed due to the potential biocidal action of hydrogen peroxide across several microorganisms, another potential limitation of these CP-containing cryogels is that they might not be able to undergo autoclave sterilization, as previously reported by our group^88^. It remains to be studied whether the highly dense and interconnected polymers in cryogels can provide sufficient protection for CP hydrolysis during autoclave treatment or whether alternative sterilization techniques could be used. Similarly, when combining CP-containing cryogels with other drugs or proteins to be released from the cryogels, possible interactions or damage resulting from the presence of hydrogen peroxide might need to be taken into consideration. Finally, the needle-injectable antibacterial cryogels reported here were 16 mm^3^. However, for some tissue engineering applications, it might be necessary to deliver larger cryogel constructs that are above the currently reported volume. In such cases, it might be best to use catheters instead of standard needle-injections.

## Conclusion

In this work, we engineered injectable microcomposite cryogels with antimicrobial properties. The cryogels with an interconnected network of large pores (10–100 µm) could be delivered using minimally invasive approaches (i.e., injected through hypodermic 16G needles) without any structural alteration. The incorporation of CP at low concentrations within the cryogels inhibited the growth of multiple pathogenic bacterial strains that are commonly associated with implant failures. The antibacterial activity seemed to be primarily driven by the production of hydrogen peroxide, a byproduct of CP degradation found in the cryogels. While hydrogen peroxide retarded or prevented bacterial growth, it had little to no effect on NIH/3T3 cell viability, and these cryogels did not seem to trigger detectable BMDC activation. Similarly, antimicrobial cryogels did not induce an inflammatory response when tested in mice even when purposely contaminated with pathogenic bacteria prior to subcutaneous injections. Furthermore, cryogels indicated clear signs of degradation in vivo. Collectively, these results indicate that needle-injectable and biodegradable CP-containing cryogels exhibit antimicrobial activity that holds great promise for a wide variety of biomedical applications, particularly when designing tissue engineering scaffolds.

## Materials and Methods

### Materials

HA (molecular weight ~ 330 kDa) was obtained from Blooming, China. Phosphate-buffered saline (PBS), dimethylformamide, glycidyl methacrylate, triethylamine, acetone, tetramethylethylenediamine (TEMED), ammonium persulfate (APS), Alizarin Red S (ARS) stain, a fluorometric hydrogen peroxide assay kit (MAK 165), bovine catalase (2000–5000 units/mg of protein), Dulbecco’s modified Eagle’s medium (DMEM), fetal bovine serum (FBS), 4′,6-diamidino-2-phenylindole (DAPI), and Triton X-100 were purchased from Sigma-Aldrich. Tryptic soy broth (TSB) was purchased from MP Biomedicals. Agar was purchased from Fisher Bioreagents. GGGGRGDSP (G4RGDSP) was synthesized by Peptide 2.0. Penicillin, streptomycin, and RPMI 1640 were purchased from Fisher Scientific. Far-red fixable live/dead stain (Alexa Fluor 647 nm) was purchased from GeneCopoeia. Alexa Fluor 488-phalloidin was obtained from Cell Signaling Technology. The BioLegend IL-6 ELISA max deluxe set (431304), BioLegend TNF-alpha ELISA max deluxe set (430904), and BioLegend IL-12 (p70) ELISA max deluxe set were used. Fluorescent antibodies for flow cytometry (specific for CD86, CD11c and MHCII) were procured from BioLegend.

### Chemical modification and characterization of HA

Methacrylate groups were added to HA to yield the HA-glycidyl methacrylate (HAGM) conjugate as per the procedure mentioned by Rezaeeyazdi et al.^103^, which allows cross-linking. Quantification of the degree of methacrylation was performed by ^1^H NMR analysis using a Varian Inova-500 NMR spectrometer. The degree of methacrylation was calculated according to a method previously described^55^. The HAGM macromonomer was found to have a degree of methacrylation of approximatively 20% (Supplementary Fig. 5).

### Fabrication and characterization of antimicrobial microcomposite cryogels

The HAGM 4% (w/v) macromonomer was dissolved in deionized water, mixed with an appropriate amount of CP, and subjected to free radical polymerization induced by 0.5% (w/v) TEMED and 0.25% (w/v) APS. This prepolymer mix was distributed into precooled Teflon molds. Polymerization was then allowed to complete at a subzero temperature, i.e., − 20 °C, for 17 h^33^. After completion of the polymerization, the gels were brought to room temperature (RT) to remove ice crystals and washed with deionized water.

The swelling ratio was determined using a standard gravimetric procedure. To calculate the swelling ratio of each sample, cryogel samples 8 mm in diameter and 6 mm in height (n = 4) were prepared and immersed in PBS at pH 7.4 and 37 °C. The equilibrium mass swelling ratio (Q_M_) was calculated by the equation Q_M_ = ms/md, where ms and md were the fully swollen gel and freeze-dried gel weights, respectively. The Young’s modulus was determined using an Instron testing system (Instron 5944). Cylindrical cryogels (6 mm in diameter, 8 mm in height) were deformed between two parallel plates with a strain rate of 10% per minute for multiple cycles. To measure the degree of interconnectivity, disc-shaped cryogels (13 mm in diameter, 1 mm in height) were fabricated. The interconnected void volume was calculated as the ratio of weight of water wicked from the gels by a Kimwipe to the wet weight of fully soaked cryogel disks^33^.

### Cryogel injectability

The ability of cryogels to pass through a conventional-gauge needle and then regain their original shapes was checked by injecting the cryogel through a hypodermic needle. First, square-shaped HAGM cryogels (dimensions: 4 mm × 4 mm × 1 mm) were suspended in 0.2 ml of PBS and syringe-injected through a 16G needle. ARS, an anthraquinone dye, has been widely used as a sensitive technique for the semi quantification of calcium deposits^63^. A 1% ARS solution was prepared in deionized water, and the pH was adjusted to 4.2. Fresh solution was used for the assay. Cryogel samples were dipped in the ARS solution for 20 min and then washed with deionized water multiple times until there was no color in the surrounding liquid.

### Microstructural imaging

SEM imaging, control (0% CaO_2_ in 4% HAGM) and antimicrobial (supplemented with 0.1% or 0.2% CaO_2_) square-shaped cryogel samples (dimensions: 4 mm × 4 mm × 1 mm) were fabricated, lyophilized, and sputter-coated with platinum to a thickness of 5 nm. Then, the cryogels were observed under a JEOL S 4800 microscope operating at a 15 kV voltage and 20 µA current. The average pore size of the cryogels was calculated by averaging the diameters of the pores as observed by SEM.

### Evaluating hydrogen peroxide release from cryogels

Hydrogen peroxide release was measured using a fluorometric hydrogen peroxide assay kit (Sigma Aldrich, MAK 165). First, square-shaped cryogel samples (4 mm × 4 mm × 1 mm) containing 0%, 0.1% and 0.2% CaO_2_ (n = 5) were placed in 200 µl of PBS per cryogel in a 24-well plate at room temperature and 200 rpm. At 15 min, 1 h, 2 h, 3 h, 4 h and 5 h, the cryogels were transferred to fresh PBS in a new well, 50 µl of the sample was removed, and the amount of H_2_O_2_ released was measured fluorometrically. Assays were performed according to the manufacturer’s instructions. The readings were normalized with the readings observed with control (0% CaO_2_) cryogels and were recorded until the fluorescence vanished.

### Assessment of antibacterial activity

Effects of calcium hydroxide, CP, and CP neutralized with catalase were tested. To evaluate the effect of individual compositions, 1 × 10^5^ MRSA cells from overnight-grown cultures were exposed to 0.1% calcium hydroxide or CP, equivalent to the amount loaded in cryogel samples. Additionally, bovine catalase (200–500 units) was used along with CP. The response in terms of cell density (i.e., absorbance at 562 nm) was monitored for 24 h and the average values were plotted (n = 5).

### Assessment of antibacterial action of CP-containing cryogels

MRSA (ATCC 43300), *P. aeruginosa* (ATCC 27853), multi-drug-resistant *E. coli* (ATCC 25922) and *Streptococcus pyogenes* (ATCC 12384) were used as test pathogens. Overnight-grown bacterial cultures were used in these experiments. Cell density was adjusted by means of absorbance to obtain 1 × 10^6^ cells/ml. Cylindrical cryogels (6 mm in diameter, 8 mm in height) with varying concentrations of CP (0%, 0.1%, and 0.2%) were sanitized, washed, partially dried over sterile gauze and inoculated with 5 × 10^4^ cells with TSB media. After 6 h of incubation at 37 °C and 5% CO_2_, the cryogels were flushed with 350 µl of sterile PBS and subjected to CFU determination using 10 µl from serial dilutions. The method has been adapted and suitably modified from^104^. For SEM imaging, samples were fixed with 4% paraformaldehyde and dehydrated serially in 20, 40, 60, 80 and 100% ethanol for 15 min each, followed by critical point drying (samdri-PVT-3D). Platinum-coated (5 nm) samples were observed with a JEOL S 4800 scanning electron microscope as mentioned earlier.

To confirm the antibacterial potential of cryogels after lyophilization as well as injection, cryogel samples (dimensions: 4 mm × 4 mm × 1 mm) with 0.2% CP were fabricated and lyophilized immediately. They were briefly sanitized, washed, partially dried over sterile gauze and inoculated with 1000 cells of bacterial pathogen in TSB. Experiments were performed with MRSA and *P. aeruginosa*. After 4 h of incubation, the cryogels were flushed with sterile PBS, and the PBS used for washing was subjected to CFU determination (Supplementary Figs. 6 and 7).

### Assessment of biocompatibility

Cryogels with 0%, 0.1% and 0.2% CP were fabricated as mentioned in previous sections. They were supplemented with 0.8% w/w ACRL-PEG-G4RGDSP to promote mammalian cell adhesion. The cryogels were briefly sanitized in 70% ethanol, washed, and partially dried over sterile gauze. The collapsed cryogels were placed in a multiwell plate. A total of 50 k 3T3 cells (NIH/3T3, CRL-1658, ATCC) suspended in 10 µl of DMEM supplemented with 10% FBS and 1% penicillin–streptomycin were uniformly distributed onto each cryogel piece. They were allowed to adhere to the cryogel for 2 h. Then, 200 µl of culture medium was added to the wells. After 24 h of incubation, the cryogels were recovered, stained with ViaQuantTM Far-red according to the manufacturer’s instructions, and fixed with 4% paraformaldehyde in PBS for 30 min. The cells were permeabilized with 0.1% Triton X-100 for 5 min in PBS and stained with Alexa Fluor 488-phalloidin and DAPI according to the manufacturer’s protocol. The cryogels were observed under a Leica TCS SP5 X WLL confocal microscope, and images were recorded. Four representative samples were used to calculate the % viability.

### Assessment of in vitro immune response with dendritic cells (DCs)

For this study, BMDCs were obtained as previously described^105^. A 24-well plate was seeded with 200 × 10^3^ cells per well in RPMI 1640 medium supplemented with FBS. Cryogels with 0%, 0.1%, and 0.2% CP of size 4 mm × 4 mm × 1 mm were used. They were briefly sanitized in 70% ethanol, washed, partially, and dried over sterile gauze, followed by 24 h of coincubation with DCs.

The supernatant was checked for the presence of secreted cytokines by ELISA. The supernatants were suitably diluted for IL-6, TNF-α, and IL-12, and assays were performed according to the manufacturer’s instructions. DCs were recovered from the well plate as well as from within the cryogels with the help of a cell scraper and were fixed with 4% paraformaldehyde treatment. The activation of DCs was assessed with flow cytometry (BD FACS Calibur DxP), and the expression levels of CD11c, CD86, and MHCII were quantified. Expression levels were interpreted based on BMDCs cultured in media alone as a negative control and BMDCs incubated in media with 100 ng/mL LPS as a positive control.

### Assessment of in vivo response

The in vivo response and tissue integration upon subcutaneous injection of CP-containing cryogels were assessed. First, cryogels (dimensions: 4 mm × 4 mm × 1 mm) were fabricated with 0% and 0.1% CP. Another set of samples consisted of CP-containing cryogels (0.1% CaO_2_) challenged with 2 × 10^3^ *P. aeruginosa* cells. The cryogels were briefly sanitized with 70% ethanol and washed with sterile PBS. Cryogels suspended in sterile PBS (0.2 mL) were syringe-injected through 16G needles in both dorsal flanks of 8-weeks old female C57BL/6J mice (n = 5/condition, The Jackson Laboratory, Bar Harbor, ME, USA)^105^. To observe cellular infiltration and signs of tissue integration, mice were sacrificed at day 4, and cryogels explanted to perform histological analysis. Cryogels were then fixed in 4% paraformaldehyde (PFA), embedded in paraffin, and then sectioned (5 μm thick). The slices were first stained with H&E or MT and subsequently imaged (Mass Histology Services, Worcester, MA, USA).

### Assessment of in vivo biodegradation

CP-free (0% CaO_2_) and 0.2% CaO_2_ cryogels (dimensions: 4 mm × 4 mm × 1 mm) were assessed for their capacity to degrade in the body. Cryogels suspended in sterile PBS (0.2 mL) were syringe-injected through 16G needles in both dorsal flanks of 12-weeks old female C57BL/6J mice (n = 5/condition, The Jackson Laboratory, Bar Harbor, ME, USA). Each mouse received both cryogel types, one on each flank. After 2 months, mice were euthanized and the cryogels explanted with the surrounding tissues to perform histological analysis. Cryogels were then fixed in 4% PFA, embedded in paraffin, and then sectioned (5 μm thick). The slices were first stained with H&E or MT and subsequently imaged (iHisto, Woburn, MA, USA). Animal work was performed under an approved protocol by the Northeastern University Standing Committee on Animals in compliance with the National Institutes of Health guidelines.

## Supplementary information

Supplementary Information 1.

Supplementary Video 1.

Supplementary Video 2.
