# Supplementary material for: Needle-injectable microcomposite cryogel scaffolds with antimicrobial properties
Source: Sci Rep. 2020 Oct 27;10:18370. doi: 10.1038/s41598-020-75196-1 (PMC7591905; doi:10.1038/s41598-020-75196-1)
Supplement: Supplementary file 1 — Supplementary Information 1. [file 41598_2020_75196_MOESM1_ESM.docx]

**Supplemental Information**

**Needle-Injectable Microcomposite Cryogel Scaffolds with Antimicrobial Properties**

Kasturi Joshi Navare^a^, Thibault Colombani^a^, Mahboobeh Rezaeeyazdi^a^, Nicole Bassous^a^, Devyesh Rana^a^, Thomas Webster^a,b^ , Adnan Memic^c^, Sidi A. Bencherif^a,d,e,f,^*

^a^ Department of Chemical Engineering, Northeastern University, Boston, MA 02115, USA;

^b^ Wenzhou Institute for Biomaterials and Engineering, Wenzhou 325001, China;

^c^ Center of Nanotechnology, King Abdulaziz University, Jeddah 21589, Saudi Arabia;

^d^ Department of Bioengineering, Northeastern University, Boston, MA 02115, USA;

^e^ Sorbonne University, UTC CNRS UMR 7338, Biomechanics and Bioengineering (BMBI), University of Technology of Compiègne, 60203 Compiègne, France;

^f^ Harvard John A. Paulson School of Engineering and Applied Sciences, Harvard University, Cambridge, MA 02138, USA

* To whom correspondence should be addressed. E-mail: s.bencherif@northeastern.edu


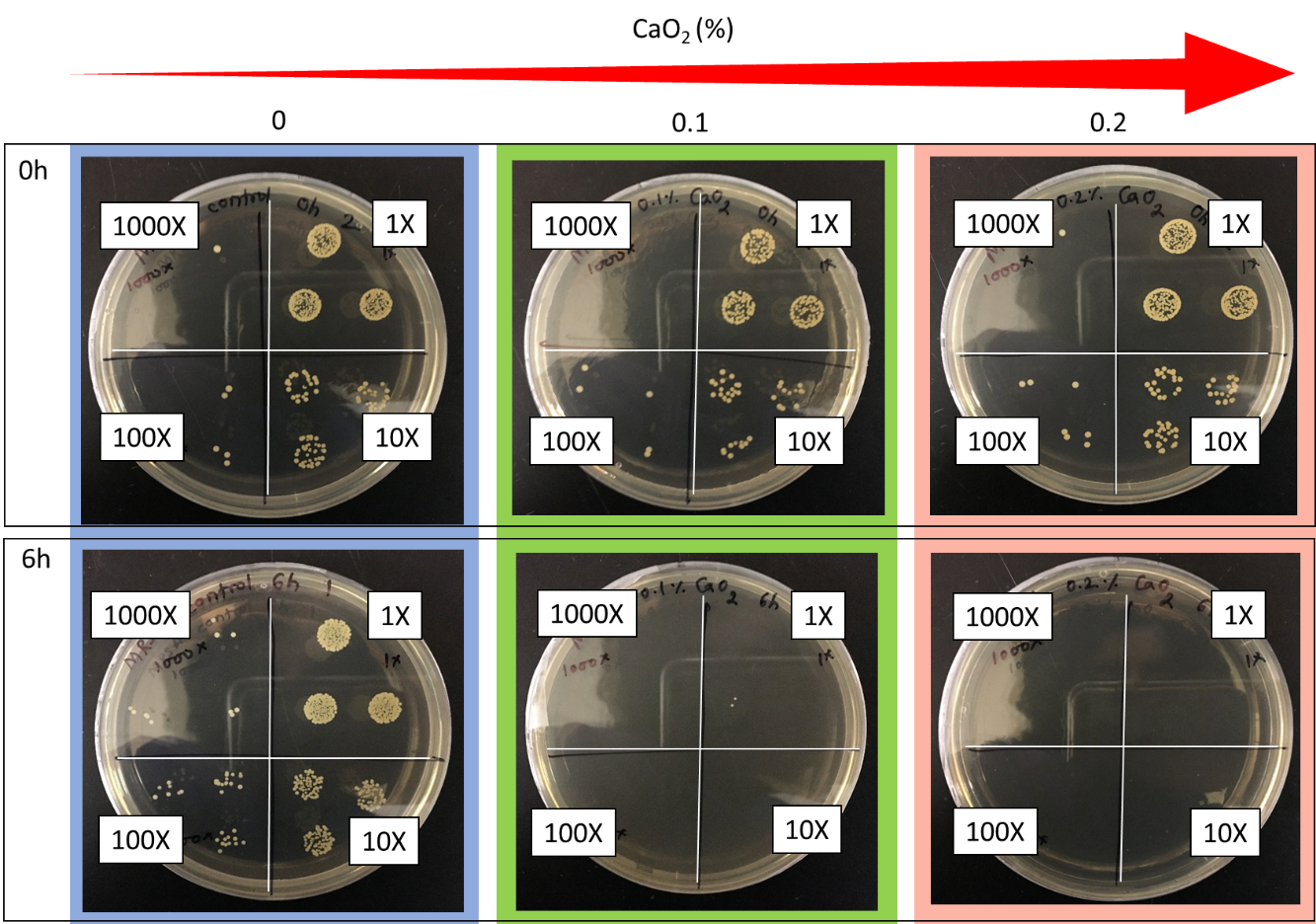


**Supplementary Figure 1. Antimicrobial cryogels prevent methicillin-resistant Staphylococcus aureus (MRSA) growth**. Antimicrobial cryogels (300µl) containing 0%, 0.1% or 0.2% (w/v) CaO_2_ were incubated with ~ 5 x 10^4^ MRSA for 0 h or 6 h. Colony Forming Units (CFUs) were then determined by extracting MRSA via PBS washing of antimicrobial cryogels then plating on tryptone soy agar plates. The PBS extract was diluted to 10X, 100X and 1000X. Pictures are representative of n= 3 samples/condition.


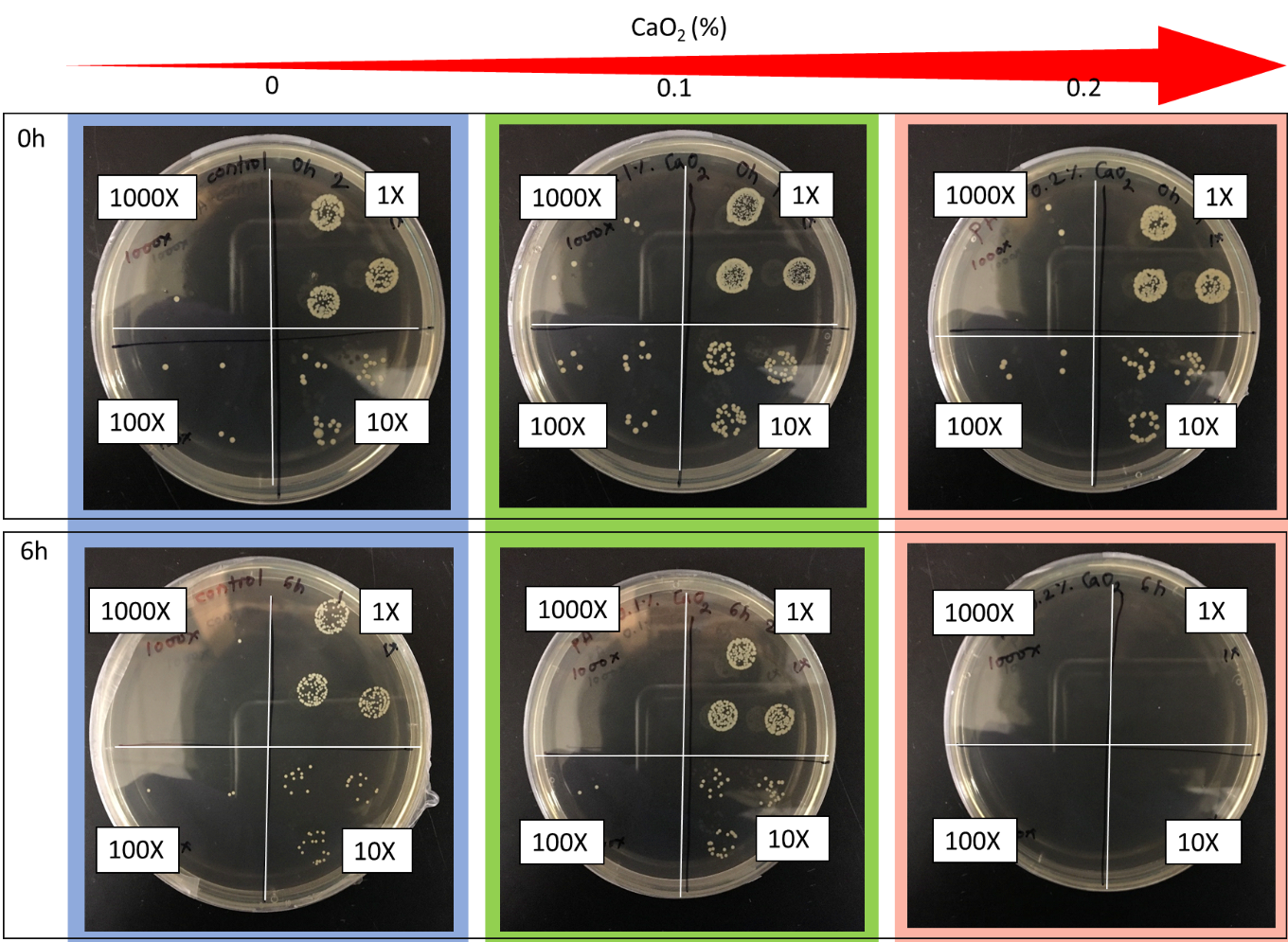


**Supplementary Figure 2. Antimicrobial cryogels prevent *Pseudomonas aeruginosa* growth**. Antimicrobial cryogels containing (300µl) 0%, 0.1% or 0.2% CaO_2_ were incubated with ~ 5 x 10^4^ *P. aeruginosa* for 0 h or 6 h. CFUs were then determined by extracting *P. aeruginosa* via PBS washing of antimicrobial cryogels then plating on tryptone soy agar plates. The PBS extract was diluted to 10X, 100X, 1000X. Pictures are representative of n= 3 samples/condition.

**Supplementary Figure 3**. **Antimicrobial cryogels prevent *Escherichia coli (E. coli)* growth.** Antimicrobial cryogels (300µl) containing 0%, 0.1% or 0.2% CaO_2_ were incubated with ~ 5 x 10^4^ *E. coli* for 6 h. CFUs were then evaluated by extracting *E. coli* via PBS washing of antimicrobial cryogels then plating on tryptone soy agar plates. The PBS extract was diluted to 10X, 100X and 1000X. Values represent mean and standard error of mean (n= 3).

**Supplementary Figure 4**. **Antimicrobial cryogels prevent *Streptococcus pyogenes* growth.** Antimicrobial cryogels (300µl) containing 0%, 0.1% or 0.2% CaO_2_ were incubated with ~ 5 x 10^4^ *S. pyogenes* for 6 h. CFUs were then evaluated by extracting *S. pyogenes* via PBS washing of antimicrobial cryogels then plating on tryptone soy agar plates. The PBS extract was diluted to 10X, 100X and 1000X. Values represent mean and standard error of mean (n= 3).


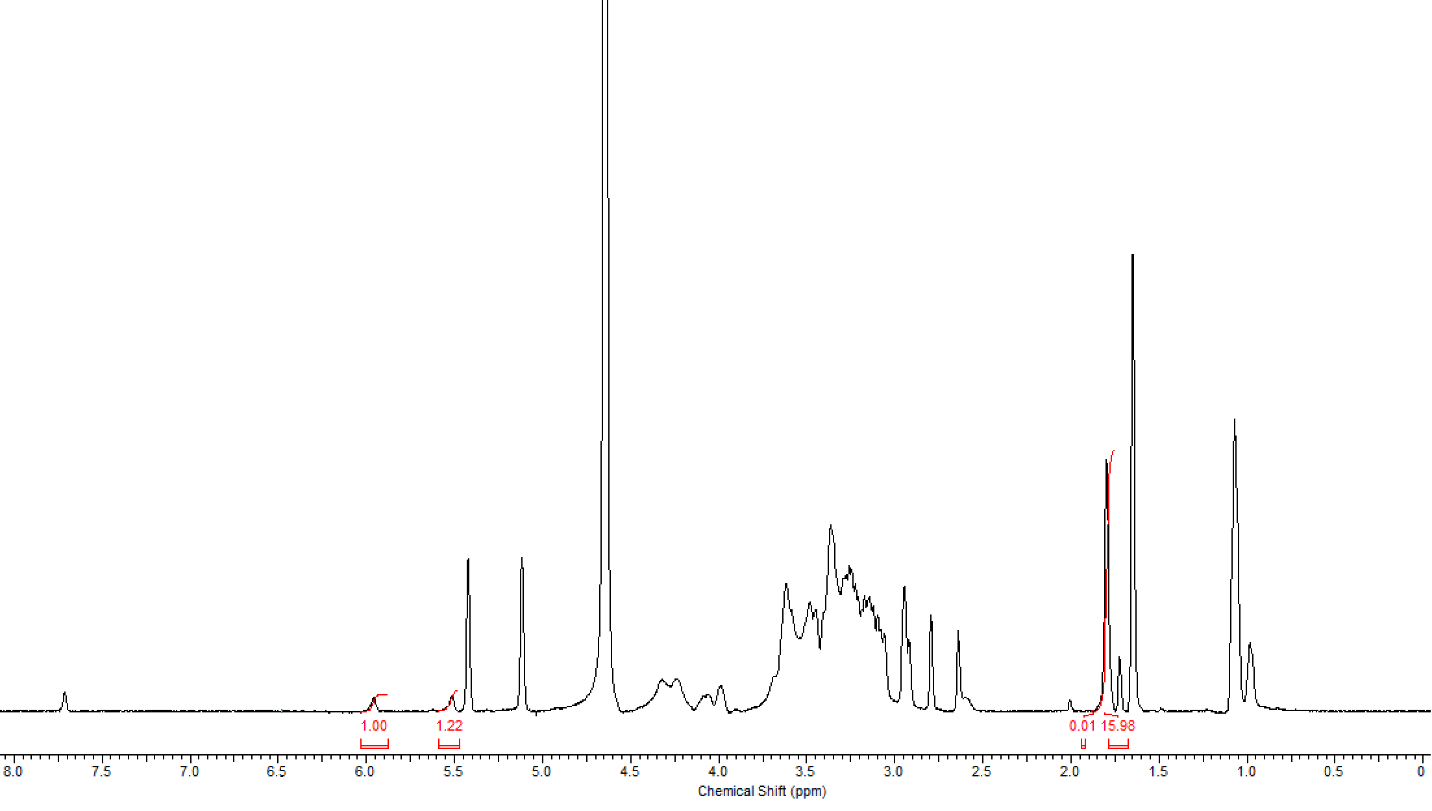


**Supplementary Figure 5. ^1^H NMR of methacrylated HA (HAGM).** Characteristic vinylic peaks were observed in the range of δ5.0-6.0 ppm. Deuterated water was used as a solvent and the degree of methacrylation was quantified to be approximately 20%.


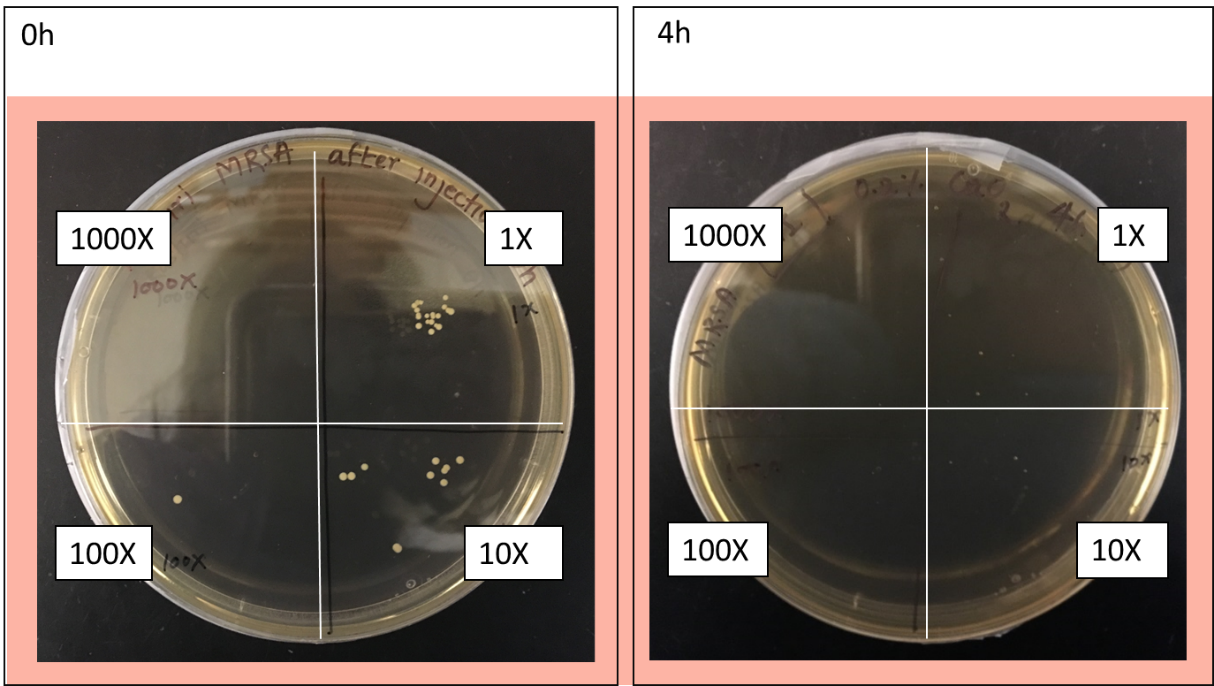


**Supplementary Figure 6. Demonstration of antimicrobial action of cryogels after injection against MRSA** Antimicrobial cryogels (16µl) containing 0.2% CaO_2_  were lyophilized, briefly sterilized with 70% ethanol, washed with PBS and incubated with 1 x 10^3^ MRSA for 4 h. CFUs were then determined by extracting MRSA via PBS washing of antimicrobial cryogels then plating on tryptone soy agar plates. The PBS extract was diluted to 10X, 100X and 1000X. Pictures are representative of n= 3 samples/condition.


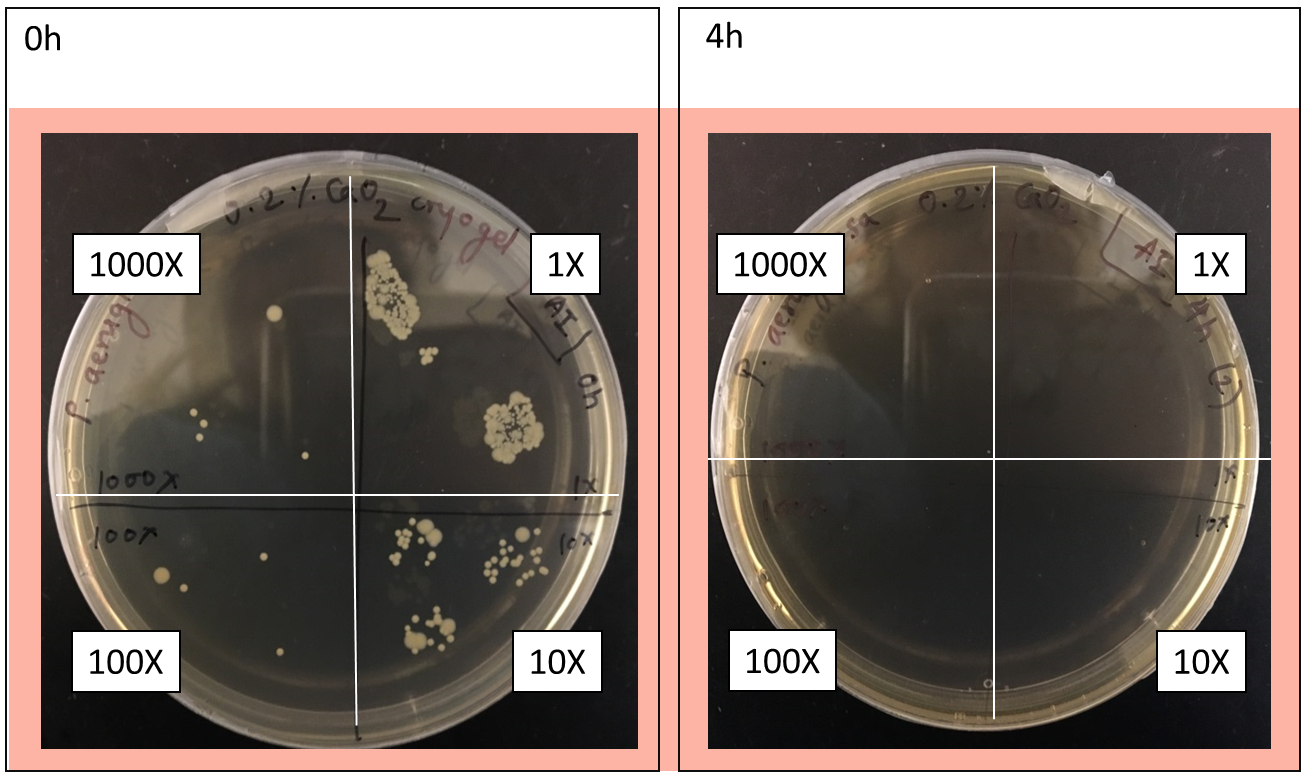


**Supplementary Figure 7. Demonstration of antimicrobial action of cryogels after injection against *P. aeruginosa*** Antimicrobial cryogels (16µl) containing 0.2% CaO_2_  were lyophilized, briefly sterilized with 70% ethanol, washed with PBS and incubated with 1 x 10^3^ *P. aeruginosa* for 4 h. CFUs were then determined by extracting *P. aeruginosa* via PBS washing of antimicrobial cryogels then plating on tryptone soy agar plates. The PBS extract was diluted to 10X, 100X and 1000X. Pictures are representative of n= 3 samples/condition.


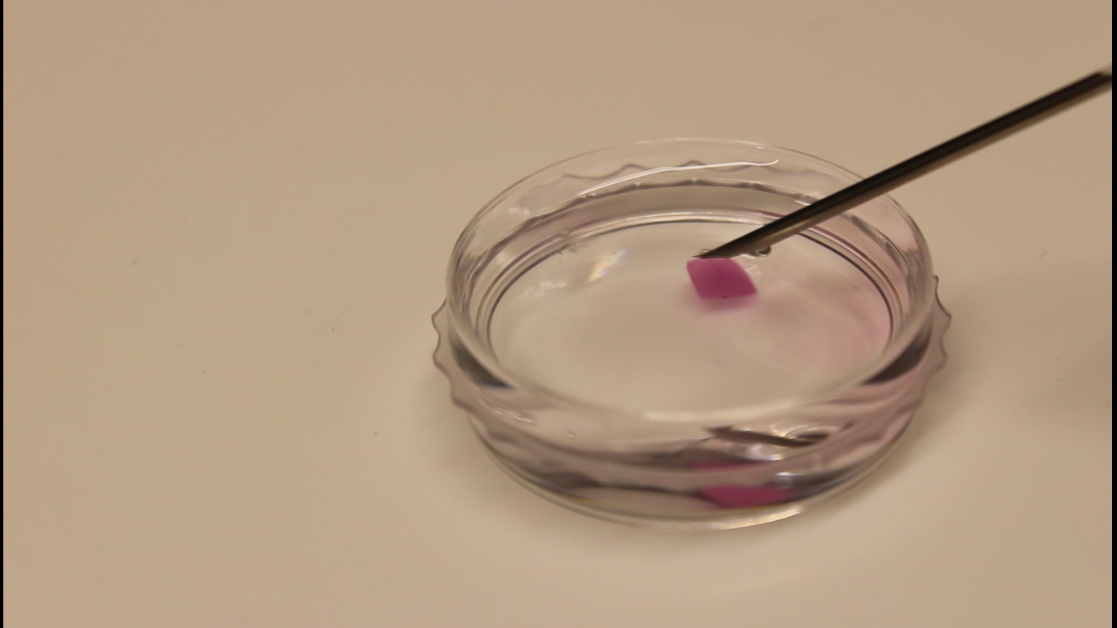


**Supplementary Movie 1. Needle-injectable CP-containing cryogels (0.1% CaO_2_).** Movie showing the successful syringe-injection through a conventional 16-gauge needle and rapid shape recovery of antimicrobial cryogels hybridized with 0.1% CaO_2_. The video is representative of n= 4 samples.


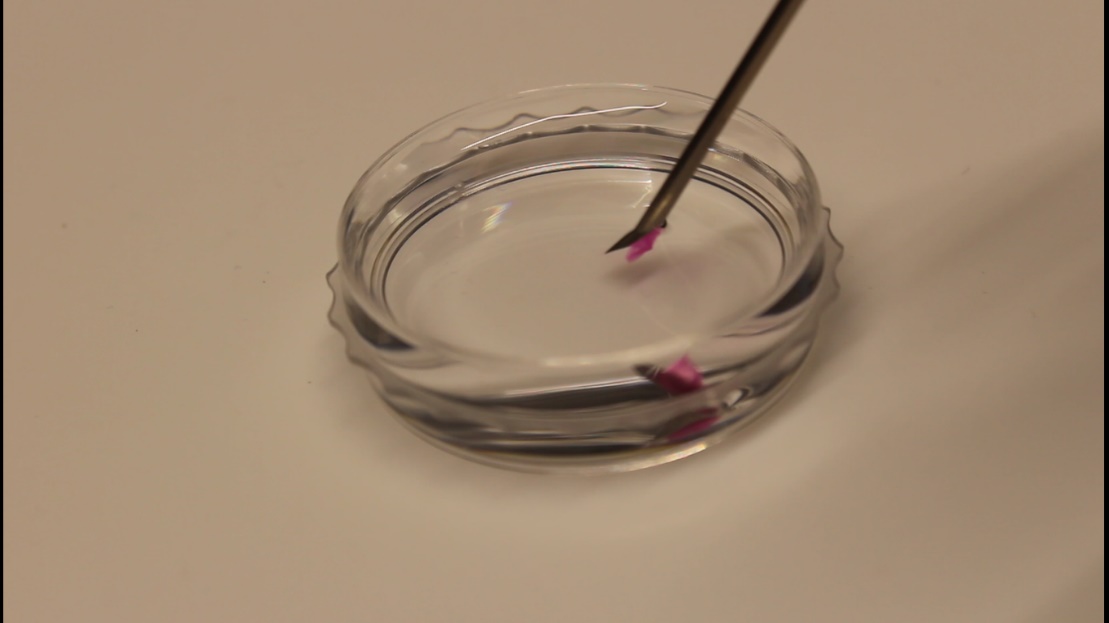


**Supplementary Movie 2. Needle-injectable CP-containing cryogels (0.2% CaO_2_).** Movie showing the successful syringe-injection through a conventional 16-gauge needle and rapid shape recovery of antimicrobial cryogels hybridized with 0.2% CaO_2_. The video is representative of n= 4 samples.
